# *PPM1D* Is a Therapeutic Target in Childhood Neural Tumors

**DOI:** 10.3390/cancers13236042

**Published:** 2021-11-30

**Authors:** Jelena Milosevic, Diana Treis, Susanne Fransson, Gabriel Gallo-Oller, Baldur Sveinbjörnsson, Nina Eissler, Keiji Tanino, Kazuyasu Sakaguchi, Tommy Martinsson, Malin Wickström, Per Kogner, John Inge Johnsen

**Affiliations:** 1 Childhood Cancer Research Unit, Department of Women’s and Children’s Health, Karolinska Institutet, 17177 Stockholm, Sweden; diana.treis@ki.se (D.T.); ggallo@alumni.unav.es (G.G.-O.); baldur.sveinbjornsson@uit.no (B.S.); nina.eissler@gmail.com (N.E.) malin.wickstrom@ki.se (M.W.); per.kogner@ki.se (P.K.); 2 Center for Regenerative Medicine, Massachusetts General Hospital, Boston, MA 02114, USA; 3 Department of Laboratory Medicine, Institute of Biomedicine, University of Gothenburg, 41345 Gothenburg, Sweden; susanne.fransson@clingen.gu.se (S.F.); tommy.martinsson@clingen.gu.se (T.M.); 4 Laboratory of Organic Chemistry II, Department of Chemistry, Faculty of Science, Hokkaido University, Sapporo 060-0810, Japan; ktanino@sci.hokudai.ac.jp; 5 Laboratory of Biological Chemistry, Department of Chemistry, Faculty of Science, Hokkaido University, Sapporo 060-0810, Japan; kazuyasu@sci.hokudai.ac.jp

**Keywords:** neuroblastoma, medulloblastoma, chromosome 17q gain, p53, WIP1, *PPM1D*

## Abstract

**Simple Summary:**

Medulloblastoma and neuroblastoma are childhood tumors of the central nervous system or the peripheral nervous system, respectively. These are the most common and deadly tumors of childhood. A common genetic feature of medulloblastoma and neuroblastoma is frequent segmental gain or amplification of chromosome 17q. Located on chromosome 17q23.2 is *PPM1D* which encodes WIP1, a phosphatase that acts as a regulator of p53 and DNA repair. Overexpression of WIP1 correlates with poor patient prognosis. We investigated the effects of genetic or pharmacologic inhibition of WIP1 activity and found that medulloblastoma and neuroblastoma cells were strongly dependent on WIP1 expression for survival. We also tested a number of small molecule inhibitors of WIP1 and show that SL-176 was the most effective compound suppressing the growth of medulloblastoma and neuroblastoma in vitro and in vivo.

**Abstract:**

Childhood medulloblastoma and high-risk neuroblastoma frequently present with segmental gain of chromosome 17q corresponding to aggressive tumors and poor patient prognosis. Located within the 17q-gained chromosomal segments is *PPM1D* at chromosome 17q23.2. *PPM1D* encodes a serine/threonine phosphatase, WIP1, that is a negative regulator of p53 activity as well as key proteins involved in cell cycle control, DNA repair and apoptosis. Here, we show that the level of *PPM1D* expression correlates with chromosome 17q gain in medulloblastoma and neuroblastoma cells, and both medulloblastoma and neuroblastoma cells are highly dependent on *PPM1D* expression for survival. Comparison of different inhibitors of WIP1 showed that SL-176 was the most potent compound inhibiting medulloblastoma and neuroblastoma growth and had similar or more potent effects on cell survival than the MDM2 inhibitor Nutlin-3 or the p53 activator RITA. SL-176 monotherapy significantly suppressed the growth of established medulloblastoma and neuroblastoma xenografts in nude mice. These results suggest that the development of clinically applicable compounds inhibiting the activity of WIP1 is of importance since *PPM1D* activating mutations, genetic gain or amplifications and/or overexpression of WIP1 are frequently detected in several different cancers.

## 1. Introduction

The mutational activation of proto-oncogenes or inactivation of tumor suppressor genes are essential processes during development of cancer [1]. The tumor suppressor gene, TP53, is one of the most commonly mutated genes in cancer. The p53 protein is a master regulator of cell growth and death by controlling DNA repair mechanisms, cell cycle progression and apoptosis [2]. Dysregulation of p53 results in genomic instability, uncontrolled cell division and inhibition of apoptosis [3,4]. While TP53 mutations are detected in more than 50% of adult cancers, pediatric cancers less often exhibit TP53 mutations [5]. Neuroblastoma and medulloblastoma are childhood tumors of the peripheral and central nervous system, respectively, that just like other childhood solid tumors infrequently harbor TP53 mutations [5]. However, p53 activity is commonly impaired in these tumors, and relapsed tumors demonstrate increased incidence of *TP53* mutations [6,7]. This suggests that inactivation of p53 is important for tumorigenesis and that alternative mechanisms for p53 attenuation are operating in these childhood cancers.

Gain of chromosome 17q is the most common chromosomal aberration and the strongest indicator of adverse outcomes in neuroblastoma [8,9,10,11,12]. Genetic analyses have shown that 75–84% of primary neuroblastoma contain either whole chromosome 17 gain or segmental gain of chromosome 17q [8,12,13,14]. In high-risk neuroblastoma, unbalanced chromosome 17q gain is detected in 90% of the patient’s tumor samples. The shortest region of chromosome 17q gain is identified as a 25 Mb-long DNA fragment (17q23.1-17qter) [13]. This chromosomal region contains several genes connected to cancers including *EME1*, *BRCA1*, *ERBB2*, *NF1*, *RAD51C*, *BRIP1*, *BIRC5* and *PPM1D*. Gain of chromosome 17q or isochromsome 17q is also the most common chromosomal aberration found in medulloblastoma [15,16,17,18]. In total, 30–50% of primary medulloblastoma contain chromosome 17q gain and patients with isochromosome 17 have earlier recurrence and worse clinical outcome [19].

Located within the gained regions of 17q in high-risk neuroblastoma and medulloblastoma is the protein phosphatase magnesium-dependent 1 delta (*PPM1D*) gene, which encodes the nuclear serine/threonine phosphatase WIP1 (wild-type p53 induced) [20,21]. WIP1 is a key regulator of p53, ATM, CHK1/2, and other molecules important in apoptosis, cell cycle progression and DNA repair, and is thus critically involved in DNA damage response and cell cycle control [22,23,24,25,26,27]. Consistently, mutations and amplifications of *PPM1D* as well as overexpression of its gene product WIP1 have been seen in a wide range of malignancies [28,29,30,31,32,33,34,35,36,37]. Additionally, studies in mice have shown that enhanced expression of *PPM1D* increases the onset of ERBB2-induced mammary gland tumors [38] and also increases the incidence of SHH-driven medulloblastomas [39]. Conversely, *PPM1D* knock-out mice demonstrated reduced frequencies of Apc-driven polyposis [40] and delayed Eµ-myc-induced B-cell lymphoma [26]. Together, these previous data suggest that *PPM1D* is important for tumorigenesis and constitutes a potential target for therapy.

## 2. Results

### 2.1. PPM1D Expression Correlates with Chromosome 17q Gains in Medulloblastoma and Neuroblastoma Cells

**Figure 1:**
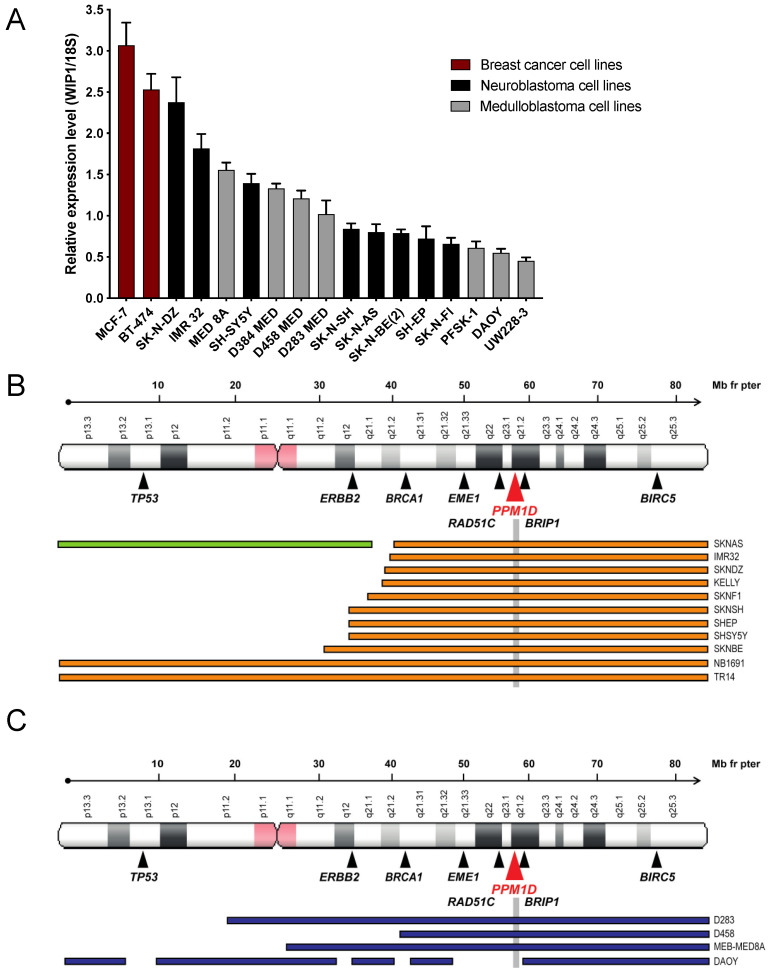
PPM1D expression correlates to 17q copy number gain. (**A**). *PPM1D* is expressed at different levels in neuroblastoma and medulloblastoma cell lines. Relative *PPM1D* mRNA expression in two *PPM1D*-amplified breast cancer cell lines (pink bars), eight neuroblastoma (NB) cell lines (black bars), six medulloblastoma (MB) cell lines (grey bars) and the supratentorial primitive neuroectodermal tumor (sPNET) cell line PFSK-1 (grey bar), analyzed with real-time PCR. The breast cancer cell line MCF-7 is amplified for *PPM1D* and used as a positive control for *PPM1D* mRNA expression. Means with S.D. of three experiments are displayed. (**B**). Chromosome 17q ploidy in neuroblastoma cell lines. Summary of whole or segmental chromosome 17 gain reported in neuroblastoma cell lines [42,43]. (**C**). Chromosome 17q ploidy in in medulloblastoma cell lines. See Figure S1 for details.

We examined the expression of *PPM1D* mRNA in a panel of cell lines, including both neuroblastoma and medulloblastoma, as well as the breast cancer cell lines MCF-7 and BT-474 exhibiting *PPM1D* gene amplification [41], and one sPNET cell line (PSFK-1) containing isochromosome 17. The cell lines expressed different levels of *PPM1D* mRNA that correlated with their genomic profile [42,43] with highest expression in *PPM1D*-amplified cell lines and lowest in those without chromosome 17 aberrations (Figure 1A,B). All neuroblastomas expressed *PPM1D* mRNA, and the highest expression was observed in SK-N-DZ and IMR32, showing expression levels comparable to the *PPM1D*-amplified breast cancer cell lines MCF-7 and BT-474 (Figure 1A). Furthermore, the relative expression level of *PPM1D* mRNA was higher in medulloblastoma cell lines with 17q-gained aberrations (D283-MED, D458-MED and MEB-MED8A) compared to cell lines with normal 17q (DAOY, UW228-3 and PFSK-1), further demonstrating a gene-dosage effect on *PPM1D* mRNA expression (Figure 1A,C).

### 2.2. PPM1D Dependency in Medulloblastoma and Neuroblastoma

**Figure 2:**
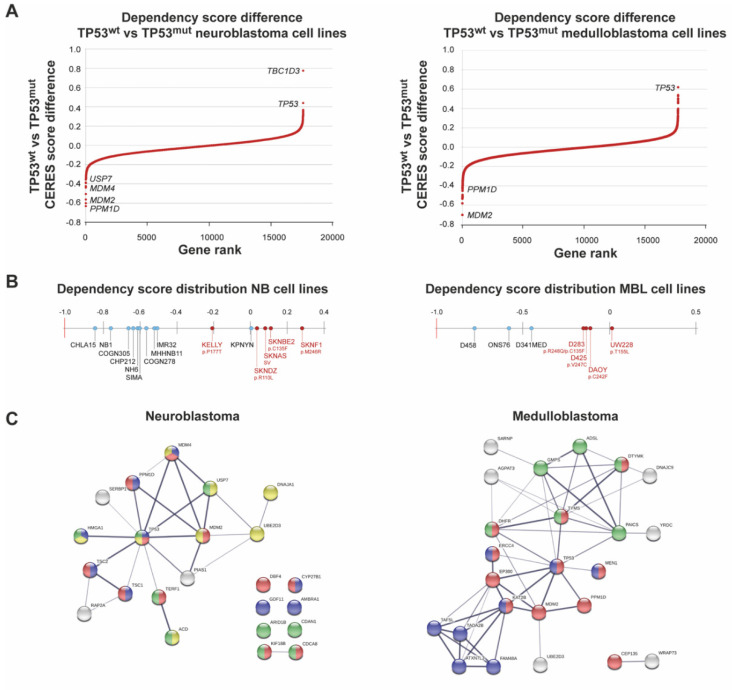

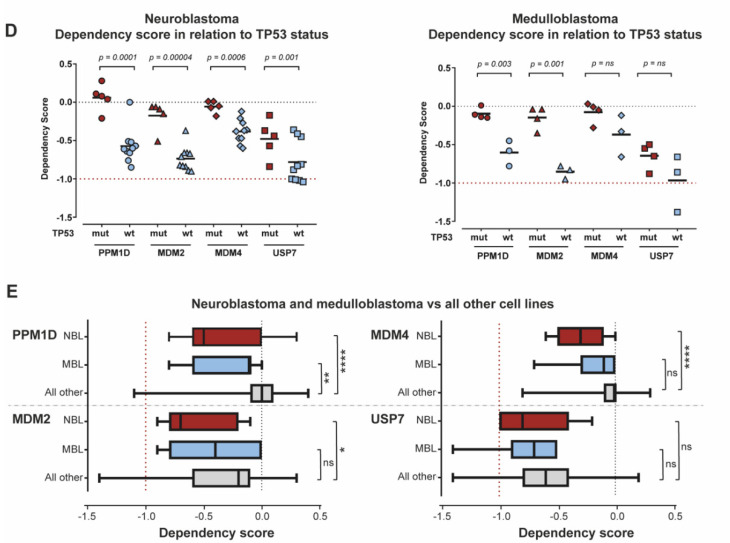
*PPM1D* expression is important for neuroblastoma and medulloblastoma survival. (**A**) Genome-scale CRISPR-Cas9 screening showing ranked average difference in genetic dependencies between wild-type *TP53* and mutated *TP53* neuroblastoma and medulloblastoma cell lines. (**B**) Dependency score showing high *PPM1D* dependency in wild-type *TP53* neuroblastoma and medulloblastoma cells. Wild-type *TP53* cell lines (blue, high dependency) and *TP53*-mutated cell lines (red, low dependency) with *TP53* status. (**C**) STRING database analysis showing *PPM1D* dependency in wild-type *TP53* neuroblastoma and medulloblastoma cells. Among the top 40 genes with the largest differences in gene dependency, as expressed as CERES scores, there was an enrichment of genes involved in the negative regulation of cell proliferation (indicated in blue), cell cycle process (red), cellular response to DNA damage (yellow) and chromosome organization (green). The width of the edges corresponds to the level of confidence (medium confidence STRING scores of 0.4; high confidence STRING score 0.7; and highest confidence STRING score 0.9). (**D**) Wild-type *TP53* neuroblastoma and medulloblastoma cells are highly dependent on *PPM1D* expression for survival. Genome-scale CRISPR-Cas9 screening showing ranked average differences in genetic dependencies between wild-type vs. *TP53*-mutated cell lines. (**E**) Dependency scores of *PPM1D*, *MDM2*, *MDM4*, and *USP7* in relation to *TP53* mutational status in neuroblastoma and medulloblastoma cell lines. For *PPM1D*, there was a statistically significantly stronger genetic dependency in neuroblastoma and medulloblastoma cells as compared with all other cell lines. * *p* < 0.05, ** *p* < 0.01, **** *p* < 0.0001.

We next used genome-scale CRISPR-Cas9 screening [44], and demonstrated that *TP53* wild-type neuroblastoma cell lines displayed a higher genetic dependency of *PPM1D* as compared to *TP53* mutated cell lines (Figure 2A,B and Table S1). Other *TP53*-repressing regulators such as *MDM2*, *MDM4* and *USP7* were ranked as number 3, 21 and 31, respectively. Among the top 40 ranked genes with the largest difference between wild-type and mutated *TP53*, an enrichment of genes involved in the negative regulation of cell proliferation (FDR = 0.0117), cell cycle process (FDR = 0.0117), cellular response to DNA damage (FDR = 0.05) and chromosome organization (FDR = 0.05) (Figure 2C) was evident.

Focusing on *PPM1D*, *MDM2*, *MDM4* and *USP7*, for all of which pharmacological inhibitors are in preclinical testing or clinical trials, we showed that neuroblastoma cell lines have preferential dependency for all four genes in *TP53* wild-type cell lines (Figure 2D). In cell lines of medulloblastoma origin, *MDM2* showed the largest difference in dependency score with negative regulators *PPM1D*, *USP7* and *MDM4,* ranked 6, 41 and 64, respectively (Figure 2A,B).

Among the top 40 genes ranked according to the largest difference between wild-type and mutated *TP53*, there is a functional enrichment of pathways associated with cell cycle (FDR = 0.000119), chromosome organization (FDR = 0.00065) and nucleotide biosynthesis processes (FDR = 0.003) (Figure 2C). As expected from the ranked differences between wild-type and mutated *TP53* medulloblastomas, only *PPM1D* and *MDM2* showed differences in genetic dependency (Figure 2D).

When comparing the genetic vulnerability of *PPM1D*, *MDM2*, *MDM4* and *USP7* in neuroblastoma and medulloblastoma cell lines to all the other cancer cell lines in the CRISPR Avana dataset, *PPM1D* showed strong selective dependency in both neuroblastoma and medulloblastoma (Figure 2E), highlighting the essentiality of intact p53.

### 2.3. Downregulation of PPM1D Expression Impairs Growth and Sensitizes Medulloblastoma and Neuroblastoma Cells to Irradiation

**Figure 3:**
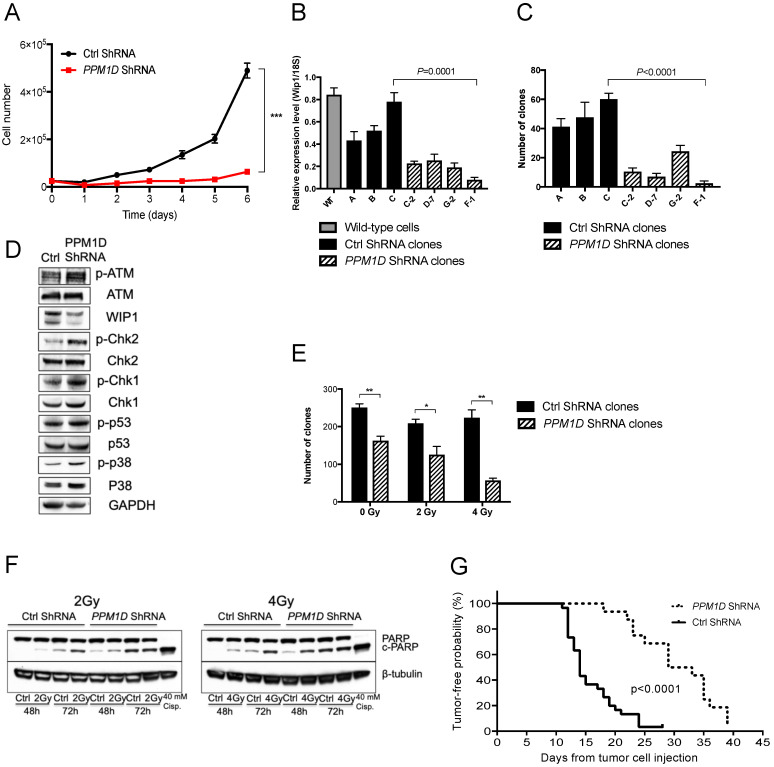
Inhibition of *PPM1D* expression suppresses tumorigenic capacity and sensitizes neuroblastoma cells to irradiation. (**A**) Knockdown of *PPM1D* with shRNA impairs proliferation of neuroblastoma cell line SK-N-BE(2). Mean with S.D. of three independent experiments is shown (*t*-test day 6, *** *p* < 0.001.) (**B**) Normalized *PPM1D* mRNA expression in wild-type SK-N-BE(2) cells (grey bar) compared to three clones transfected with a non-silencing control shRNA (black bars, **A**–**C**) and four different *PPM1D* shRNA knockdown clones (hatched bars, C-2, D-7, G-2 and F-1). The expression of *PPM1D* mRNA was significantly lower in the clones transfected with the different *PPM1D* shRNAs compared to the control transfected clones (one-way ANOVA with Bonferroni correction *p* < 0.05). Mean with S.D. of three determinants is shown. (**C**) Knockdown of *PPM1D* inhibits colony-forming ability of neuroblastoma cells. Clonogenic assay of SK-N-BE(2) cells showing decreased colony formation in shRNA *PPM1D* knockdown cells (one-way ANOVA with Bonferroni post-test *p* < 0.0001) with lowest colony-forming ability in the F-1 clone (*t*-test *p* < 0.0001). Mean with S.D. of nine determinants is displayed. (**D**) Knockdown of *PPM1D* inhibits dephosphorylation of WIP1 target genes. Phosphorylation levels of WIP1 targets increased after *PPM1D* knockdown (clone F-1) compared to control transfected cells (clone C), as shown by Western blotting. (**E**) *PPM1D* downregulation sensitizes neuroblastoma cells to irradiation. *PPM1D* knockdown of SK-N-BE(2) cells showed an irradiation dose-dependent decrease in clonogenic capacity compared to control transfected cells. Mean with S.D. of three experiments (*t*-test, * *p* < 0.05, ** *p* < 0.01). (**F**) ShRNA-mediated knockdown of *PPM1D* increases irradiation-induced apoptosis. Protein expression of the pro-apoptotic marker cPARP in *PPM1D* knockdown cells compared to control transfected cells 48 and 72 h after exposure to irradiation, analyzed by Western blot. β-tubulin was used as protein loading control. Cisplatin was used as positive control. (**G**) Knockdown of *PPM1D* delays neuroblastoma development. Clone F-1 and control clone C were injected subcutaneously in NMRI *nu/nu* mice (shRNA, *n* = 8; control, *n* = 15), 5 million cells bilaterally. Tumor development was significantly delayed (log-rank test *p* < 0.0001) showing median time to tumor development (defined as tumor volume ≥ 0.1 mL) to be more than doubled (33 days vs. 15 days) after *PPM1D* downregulation (dashed line) compared to animals injected with cells transfected with the non-silencing control shRNA (black line).

To study the potential tumorigenic function of *PPM1D*/WIP1 in neuroblastoma and medulloblastoma, we next used genetic manipulation through the stable knockdown of short-hairpin RNA (shRNA) against *PPM1D*. The majority of neuroblastoma and medulloblastoma cell lines transfected with *PPM1D* shRNA were non-viable compared to scrambled controls (data not shown), whereas cells that were viable after transfection showed reduced proliferation and increased H2AX phosphorylation, suggesting an effect on cell viability and genomic integrity (Figure 3A and Figure S2). The multi-resistant neuroblastoma cell line SK-N-BE(2), isolated from a patient with recurrent stage 4 neuroblastoma, was chosen for further analysis, since this was one of the few cell lines that was viable after *PPM1D* shRNA knockdown.

Analyzing different shRNAs constructs directed against *PPM1D* mRNA in SK-N-BE(2) cells established *PPM1D*-shRNA F-1 as the most effective in reducing *PPM1D* mRNA levels (Figure 3B). In concordance, all *PPM1D*-shRNA clones displayed impaired clonogenic capacity compared to the control shRNA clones (Figure 3C). *PPM1D* knockdown was also confirmed by increased phosphorylation of WIP1 target proteins including ATM, CHK1, CHK2, p53 and p38, all involved in cell cycle regulation and the DNA-damage response (DDR) (Figure 3D and Figure S4).

To test the role of *PPM1D* in cellular stress, we next subjected SK-N-BE(2) cells to irradiation, and showed that cells lacking *PPM1D* exhibited increased sensitivity to irradiation compared to control cells (Figure 3E and Figure S4). Moreover, higher levels of PARP cleavage were detected in *PPM1D* shRNA-transfected cells compared to controls, suggesting an increased vulnerability to apoptosis following irradiation (Figure 3F).

Furthermore, to evaluate the effect of *PPM1D* knockdown on tumor development in vivo, SK-N-BE(2) cells with either *PPM1D*-knockdown or control shRNA were injected subcutaneously in mice and tumor growth was compared. Tumor development was significantly delayed with median time to tumor development (≥0.1 mL tumor volume) more than doubled (33 days median, vs. 15 days) in the *PPM1D*-shRNA knockdown group compared to animals injected with control shRNA-transfected cells (*p* < 0.001) (Figure 3G).

### 2.4. Pharmacological Targeting of WIP1 Impairs Medulloblastoma and Neuroblastoma Growth

Having established that *PPM1D*-knockdown reduces tumor formation in mice and that gain of chromosome 17q harboring *PPM1D* is a strong prognostic factor for poor survival in both neuroblastoma and medulloblastoma [45,46], we next investigated the effects of compounds inhibiting WIP1 activity in both cancers.

**Figure 4:**
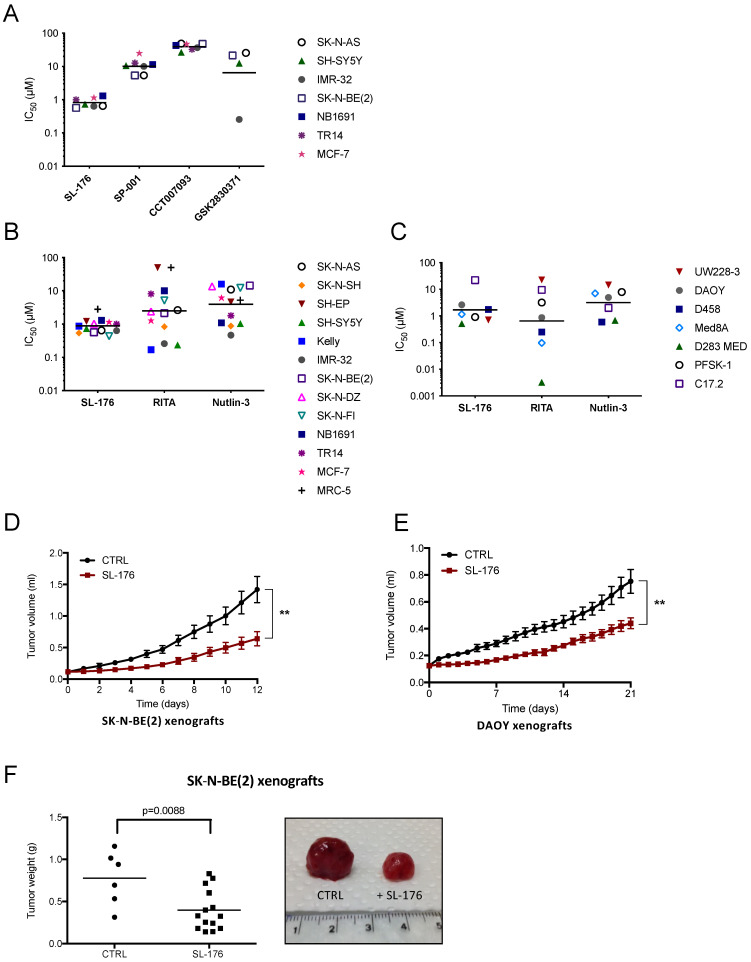

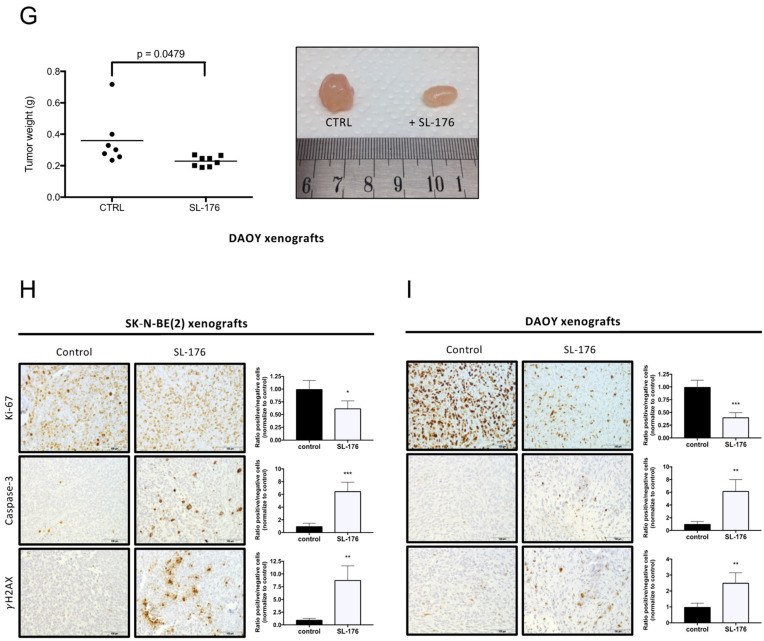
The WIP1 phosphatase inhibitor SL-176 suppresses neuroblastoma and medulloblastoma growth in vitro and in vivo. (**A**) SL-176 is more or equally efficient in inhibiting tumor cell viability among the WIP1 inhibitors tested. IC_50_ values for four to six neuroblastoma cell lines and the *PPM1D*-amplified breast cancer cell line MCF-7 exposed to the WIP1 inhibitors SL-176, SPI-001, CCT007093 and GSK2830371. Horizontal lines indicate mean. IC_50_ values were calculated from cell viability assays performed at least three times. SL-176 displayed a lower mean of IC_50_ values than SPI-001 and CCT007093, while no difference was observed when comparing with GSK2830371 (mixed effects model on log IC_50_ *p* < 0.0001, Bonferroni post-test: SL-176 vs. SPI-001 *p* < 0.0001, SL-176 vs. CCT007093 *p* < 0.0001, SL-176 vs. GSK2830371 *p* = 0.74). (**B**) SL-176 is an efficacious inhibitor of neuroblastoma cell viability. IC_50_ values for eleven neuroblastoma cell lines, the breast cancer cell line MCF-7 and the fibroblast cell line MRC-5 exposed to the specific WIP1 inhibitor SL-176, the p53–MDM2 interaction inhibitor RITA or the MDM2 antagonist Nutlin-3. Horizontal lines indicate mean. SL-176 displayed the lowest mean of IC_50_ values in the neuroblastoma cell lines of the three tested compounds (mean IC_50_ for SL-176: 0.77 µM; RITA: 2.0 µM; and Nutlin-3: 3.7 µM); however, a significant difference was only evident between SL-176 and Nutlin-3 (repeated measures one-way ANOVA on log IC_50_ *p* = 0.030, Bonferroni post-test SL-176 vs. Nutlin-3 *p* = 0.0088, SL-176 vs. RITA *p* = 0.14). IC_50_ values were calculated from the results of the cell viability assay WST-1 performed at least three times. (**C**) SL-176 inhibits medulloblastoma cell viability. IC_50_ values for five medulloblastoma cell lines, the supratentorial primitive neuroectodermal (sPNET) tumor cell line PFSK-1 and the murine neural progenitor cell line C17.2 exposed to the specific WIP1 inhibitor SL-176, the p53–MDM2 interaction inhibitor RITA or the MDM2 antagonist Nutlin-3. Horizontal lines indicate mean. No significant differences between the mean IC_50_ values were shown in these medulloblastoma cell lines (mean IC_50_ for SL-176: 1.3 µM, RITA: 1.1 µM and Nutlin-3: 4.7 µM, repeated measures one-way ANOVA *p* = 0.10). IC_50_ values were calculated from the results of the cell viability assay WST-1 performed at least three times. MRC-5 and C17.2 were used as a non-tumorigenic control for drug toxicity. (**D**,**E**) SL-176 inhibit neuroblastoma (**D**) and medulloblastoma (**E**) growth in vivo. Nude mice were injected with neuroblastoma SK-N-BE(2) cells to form xenografts on the flank. Daily i.p. injections of SL-176 (*n* = 14) for 12 days, starting at tumor volume 0.1 mL, compared to no treatment (CTRL, *n* = 6) showed that WIP1 inhibition through SL-176 significantly impaired the growth of neuroblastoma xenografts (*t*-test day 12, *p =* 0.002). Nude mice engrafted with medulloblastoma DAOY xenografts were treated from tumor volume 0.12 mL, receiving either daily i.p. injections of SL-176 (*n* = 8) for 21 days or no treatment (*n* = 7). SL-176 treatment significantly delayed medulloblastoma xenograft growth (*t*-test day 21, *p* = 0.0051). Mean with S.E.M. (**F**,**G**) Tumor weight at autopsy of SK-N-BE(2) (**F**) and DAOY (**G**) xenografts and representative photographs of dissected neuroblastoma and medulloblastoma xenograft tumors in comparison. (**H**,**I**) SL-176 decreases proliferation, induces apoptosis and activates γH2AX in xenograft tumors. Immunohistochemical analysis of SK-N-BE(2) (**H**) and DAOY (**I**) xenograft tumors. Tumor sections were stained with anti-Ki-67, anti-Caspase 3, and anti-γH2AX antibodies. Representative examples of immunostainings are shown. Images were acquired at 400× magnification. Identification and quantification of positive and negative cells was carried out with ImageJ software (*t*-test, * *p* < 0.05, ** *p* < 0.01, *** *p* < 0.001).

To assess the cytotoxic effects of different WIP1 inhibitors on cell viability, six neuroblastoma cell lines and the *PPM1D*-amplified breast cancer cell line MCF-7 were exposed to different concentrations of the WIP1 inhibitors SL-176, SPI-001, CCT007093 or GSK2830371 [47,48,49,50,51]. Treatment of neuroblastoma cell lines with the specific WIP1-inhibitor SL-176 achieved complete inhibition with IC_50_ values within a rather narrow range of 0.57–1.3 µM, regardless of p53 or MDM2 status (Figure 4A, Figure S3A and Table S2A). The WIP1 inhibitors SPI-001 and CCT007093 did not reach complete inhibition at the concentrations tested, and the IC_50_ values spanned from 1.0 to >25 µM and 27 to 48 µM, respectively. Additionally, for these two WIP1 inhibitors, sensitivity of neuroblastoma cells did not cluster according to p53 or MDM2 status. For the WIP1 inhibitor GSK2830371 the IC_50_ values ranged from 0.25 to 26 µM, where the p53 wild type (SH-SY5Y) was the most sensitive and the p53 mutated cell lines (SK-N-AS, SK-N-BE(2)) showed more resistance. Among the WIP1 inhibitors tested, SL-176 displayed the lowest mean IC_50_, with a significantly lower IC_50_ than SPI-001 and CCT007093 (*p* < 0.001); however, no significant difference was observed between the mean IC_50_ values of SL-176 and GSK2830371.

To compare the effect of WIP1 inhibition with the effect of alternative indirect p53 reactivation, a panel of eleven neuroblastoma and six medulloblastoma/sPNET cell lines were treated with different concentrations of SL-176, the p53 modulator RITA, and the MDM2 antagonist Nutlin-3. The breast cancer cell line MCF-7 and two non-cancerous cell lines—MRC-5 derived from human fetal lung fibroblasts and C17.2 derived from murine neural progenitor cells—were also included in the screening (*TP53* status is shown in Table S3). SL-176 again displayed a narrow span of IC_50_ values of 0.44–1.3 µM and was more efficient than Nutlin-3 and as potent as RITA against the neuroblastoma cell lines (mean IC_50_ for SL-176: 0.77 µM, RITA: 2.0 µM and Nutlin-3: 3.7 µM) (Figure 4B, Figure S3B and Table S2B). Neuroblastoma cell line sensitivity toward RITA spread between IC_50_ values 0.19 and >50 µM without any apparent clustering according to known cell line genetics, while sensitivity toward Nutlin-3 tended to be greater among *TP53* wild-type neuroblastoma cell lines, with IC_50_ ranging between 0.47 and 16 µM (Figure 4B, Figure S3B and Table S2B).

For medulloblastoma and sPNET cell lines, SL-176 had similar effects on viability as the p53 inhibitors RITA and Nutlin-3. However, no significant differences between the mean IC_50_ values in medulloblastoma cell lines were observed (mean IC_50_ for SL-176: 1.1 µM, RITA: 0.41 µM and Nutlin-3: 3.4 µM; *p* = 0.26) (Figure 4C, Figure S3B and Table S2B). All three drugs achieved complete inhibition of most cell lines tested (Figure S3B). The non-cancerous cell lines MRC-5 and C17.2 were less sensitive to SL-176 than the cancer cell lines (IC_50_ for SL-176: 2.8 and 22 µM, RITA: >50 and 9.5 µM and Nutlin-3: 5.3 and 1.0 µM) (Figure 4B,C, Figure S3B and Table S2B).

Having established SL-176 as the most potent inhibitor of the WIP1–MDM2–p53 circuit among the compounds tested, we next investigated the anti-tumorigenic effect of SL-176 monotherapy in preclinical in vivo models of neuroblastoma and medulloblastoma. Tumor growth inhibition was observed after one day of treatment (SK-N-BE(2); *p* = 0.01, DAOY; *p* = 0.02) (Figure 4D,E). Both tumor volume and weight at the end of the experiment were significantly smaller in SL-176-treated mice compared to control mice (SK-N-BE(2); *p* < 0.01, DAOY; *p* < 0.05) (Figure 4F,G). No adverse effects of SL-176 were observed in the treatment groups. Histologically, SL-176-treated tumors showed an increase in active caspase-3 and phosphorylation of the DNA repair protein γH2AX, whereas reduced levels of the proliferation marker Ki-67 were observed (Figure 4H,I).

## 3. Discussion

Neuroblastoma and medulloblastoma are childhood tumors of the developing peripheral and central nervous system, respectively, which despite intensified multimodal therapy still have a poor outcome when compared to pediatric cancers in general. In order to improve management, clinical care and the chance of a cure for these patients, a detailed molecular understanding of the diseases and the development of new therapies based on this understanding are essential. A common genetic feature of neuroblastoma and medulloblastoma is segmental gain of chromosome 17q, which in both diseases is a predictor of poor prognosis [8,45,52]. Frequent gain of chromosome 17q is also observed in cancers with epithelial, neural or hematopoietic origin [53,54,55,56,57,58]. This suggests that one or multiple genes important for tumorigenesis are located on 17q. Several cancer-associated genes have been identified on chromosome 17q, including *PPM1D*, *EME1*, *BRCA1*, *ERBB2*, *NF1*, *RAD51C*, *BRIP1* and *BIRC5*. Given the importance of *PPM1D* as a key regulator of cellular responses to DNA damage, as well as the frequent detection of gene mutations, gains or amplifications of *PPM1D* in various cancers resulting in the overexpression of WIP1 or the expression of truncated, oncogenic versions of WIP1 proteins [33,37,59,60], *PPM1D* stands out as a strong candidate for tumorigenic involvement.

To functionally test the importance of *PPM1D* in neuroblastoma and medulloblastoma, we investigated the effects of genetic or pharmacological inhibition of WIP1 and demonstrated that blocking the expression or activity of WIP1 suppressed both neuroblastoma and medulloblastoma growth in vivo. These findings, together with similar observations [34,61,62], further support that WIP1 is important for the development and progression of these neural tumors. WIP1 is a homeostatic regulator of the DNA damage response (DDR) cascade by dephosphorylation and inactivation of ATM, ATR, CHK1, CHK2 and DNA-dependent protein kinase catalytic subunit [63,64]. Expression of WIP1 is directly guided by p53 acting as a transcription factor on elements within the 5′-untranslated region of the *PPM1D* gene [20]. This autoregulatory loop results in WIP1-mediated dephosphorylation of p53 (Ser15) and p53 inactivation. WIP1 also dephosphorylates and inactivates the p53-activating kinases ATM, CHK1 and CHK2, which phosphorylate p53 at Ser15 and Ser20, respectively [22,23,24,26,65]. These dephosphorylation events allow MDM2 to interact with p53 and mediate proteasomal degradation [38]. This and concurrent WIP1-mediated inactivation of p53 will reduce the fidelity of overall DNA repair mechanisms and promote the accumulation of DNA aberrations, which is a prerequisite for tumorigenesis [5]. Accordingly, we observed increased phosphorylation of DDR proteins and H2AX, enhancing the sensitivity to irradiation of *PPM1D* knockdown neuroblastoma cells. Additionally, phosphorylation of p53 (Ser15) was increased in *PPM1D* knockdown cells. Although mutations of *TP53* are not commonly detected at time of diagnosis in neuroblastoma or medulloblastoma, the p53 activity is recurrently compromised in these tumors [52,66,67,68,69], and p53 inactivation has been shown to contribute significantly to neuroblastoma and medulloblastoma development in specific animal models [70,71,72]. Our results indicate that p53 activity is restored in cells expressing low or no WIP1. We also tested the anti-tumorigenic effect of four WIP1 inhibitors, SL-176, SPI-001, CCT007093 and GSK2830371 [47,48,49,50,51], as well as the p53 modulators RITA and Nutlin-3 [73,74]. Among these compounds, SL-176 was the most universally potent compound in inducing cytotoxicity across different neuroblastoma cell lines and had cytotoxic effects similar to RITA and Nutlin-3 in medulloblastoma cells.

The WIP1 inhibitor SL-176 was developed as a simplified analog of SPI-001 and has been shown to inhibit the phosphatase activity of WIP1 by noncompetitive inhibition [49,51]. SL-176 displays an inhibitory profile which deviates from the one seen in the allosteric WIP1 inhibitor GSK2830371: while the latter exhibits its inhibitory effect only in sensitive cell lines with wild-type *TP53* [61,75], SL-176 affects the viability of virtually all tested NB and MB cell lines with IC_50_ values around 1 µM, regardless of *TP53* mutational status, while non-cancerous cell lines show much lower sensitivity (Figure 4A and Figure S2B) [76]. Admittedly, this stands in contrast to our DepMap findings, where gene dependency on *PPM1D* clusters to *TP53*-mutated cell lines. On the other hand, this comprehensive effect is concordant with the results of our *PPM1D* knockdown results, which prove that *PPM1D* is essential for NB proliferation even in the *TP53*-mutated cell line SK-N-BE(2), in vitro and in vivo. Moreover, Ogasawara et al. already showed that SL-176 is effective against the NSCLC cell line NCI-H1299, which lacks p53 expression [49].

Additionally, the p53 modulator RITA—in contrast to Nutlin-3—exhibited its effect independently of the *TP53* mutational status of the neuroblastoma cell lines studied. This finding is consistent with previous observations and it has been suggested that this might be explained by a conformational change in p53 that also pertains to the mutated protein [77]. Thus, differences between susceptibility according to *TP53* mutational status have been seen both in p53 modulators and WIP1 inhibitors. From a clinical point of view, it is encouraging that WIP1 inhibition can be effective even in *TP53*-mutated tumors, given that *TP53* mutations are often prevalent in recurrent and refractory neuroblastomas. Aberrant expression of *PPM1D* caused by chromosomal gains, gene amplification or activating mutations has been described in multiple cancers and high expression of *PPM1D* often correlates with poor patient outcome [78]. Moreover, high expression and/or genetic aberration of *PPM1D* is frequently found in cancers with wt*TP53*, suggesting that high protein levels or stability of WIP1 inhibits the activity of p53, which can result in neoplastic transformation and malignant tumor formation [79,80]. These data, together with the demonstration that *PPM1D*-deficient mice show a delayed onset of mammary gland tumor development, whereas overexpression of *PPM1D* in mice subjected to external DNA stress develop cancers that are highly similar to tumors in p53-deficient mice [81], strongly suggest that *PPM1D* is an oncogene. Genetic analyses have shown the presence of unbalanced chromosome 17q gain in 90% of high-risk neuroblastomas, and high expression of *PPM1D* is associated with adverse patient outcome [8,12,13,14,34]. Hence, the majority of high-risk neuroblastoma patients could potentially have benefits from treatment including a WIP1 inhibitor. Chromosome 17q gain or isochromsome 17q is also the most common chromosomal aberration found in medulloblastoma. A total of 30–50% of primary medulloblastoma contain chromosome 17q gain. The highest expression of *PPM1D* is observed in Group 3 and Group 4 as well as in metastatic medulloblastomas [28]. *PPM1D* expression is also associated with worse overall and progression-free survival in patients with these tumors [28,82]. Therefore, WIP1 inhibitors could have potential as a treatment option in Group 3 and Group 4 medulloblastoma patients as well as in SHH patients with wt*TP53* expressing high levels of *PPM1D*.

Here, we show that WIP1 constitutes a druggable target in neuroblastoma and medulloblastoma that should be further developed and evaluated in combination with current treatment modalities and investigated for testing in clinical trials, given the fact that the majority of patients with poor prognosis have aberrant expression of WIP1 [34,82]. It may be argued that targeting DNA repair mechanisms and phosphatase activity in particular seems a problematic hurdle, but our current data are promising proofs of a principle providing molecular and pharmacological evidence. Furthermore, genetic instability and accumulation of genetic aberrations over time are major obstacles in metastatic and relapsing pediatric cancers, further supporting the potential role and impact of WIP1 as a promising therapeutic target for pediatric patients with high-risk neuroblastoma and medulloblastoma, as well as a wide range of adult cancer patients.

Overexpression of *PPM1D* promotes the growth and treatment resistance of pediatric and adult cancers [19]. Our study together with others has shown that dysregulation of WIP1 is targetable with small-molecule inhibitors [19]. Additionally, compared with conventional chemotherapies, interventions that modulate the activity of WIP1 could provide a more specific option with reduced cytotoxicity. Hence, compounds that inhibit the activity of WIP1 either directly or indirectly should have potential as a treatment option in patients with wt*TP53* and aberrant WIP1 expression.

## 4. Materials and Methods

### 4.1. Cell Culture and Reagents

Twenty-two human cell lines of different origin were used throughout the study: eleven neuroblastoma cell lines (IMR-32, Kelly, NB1691, SH-EP, SH-SY5Y, SK-N-AS, SK-N-BE(2), SK-N-DZ, SK-N-FI, SK-N-SH, TR14), eight medulloblastoma/sPNET cell lines (DAOY, D283MED, D384MED, D425MED, D458MED, MEB-MED8A, PFSK-1, UW228-3), two breast cancer cell lines (MCF-7, BT-474), and one human fetal lung fibroblast cell line (MRC-5). In addition, one neural multipotent progenitor cell line from mouse (C17.2) was used. The cell lines were purchased from ATCC, except D384MED, D425MED, D458MED, PFSK-1, MEB-MED8A and UW228-3, which were kindly provided by Dr. M. Nistér (Karolinska Institutet), NB1691 and TR14 by Dr. D. Tweddle (Wolfson Childhood Cancer Research Centre, Newcastle University, Newcastle upon Tyne, NE1 7RU, UK) and C17.2 by Dr. T. Ringstedt (Dept. Woman´s and Children´s Health, Karolinska Institutet, 171-77 Solna, Sweden).

The cell lines were cultured in RPMI 1640 (IMR-32, Kelly, NB1691, SH-EP, SK-N-AS, SK-N-BE(2), SK-N-DZ, SK-N-FI, SK-N-SH, TR14, PFSK-1 and MRC-5), Dulbecco’s modified Eagle’s medium (DMEM; MEB-MED8A, C17.2, BT-474), Minimum Essential Media (MEM; DAOY, D283MED, D384MED), Richter’s improved MEM with zinc/DMEM (IMEMZO/DMEM; D425MED and D458MED), or DMEM/F12 (SH-SY5Y and UW228-3). Medium was supplemented with 10% (or 15% for C17.2, MEB-MED8A, D425MED and 20% for D384MED) heat-inactivated fetal bovine serum (FBS), 2 mM L-glutamine, 100 IU/mL penicillin G, and 100 μg/mL streptomycin (Life Technologies Inc., Stockholm, Sweden) at 37 °C in a humidified 5% CO_2_ atmosphere. To the MCF-7 and D384MED media, 1 mM sodium pyruvate and 1 mM non-essential amino acids solution (Gibco) were also added. All media were purchased from Gibco BRL.

The identities of the cell lines were verified by short tandem repeat genetic profiling using the AmpFlSTR^®^ IdentifilerTM PCR Amplification Kit (Applied Biosystems) in December 2015 and all cell lines were used in passages below 25. All experiments were executed in Opti-MEM (GIBCO) supplemented with glutamine, streptomycin and penicillin (HyClone Thermo Fisher Scientific, Waltham, MA, USA), except transfection experiments, which were performed without antibiotics.

*PPM1D*-knockdown SK-N-BE(2) cells and corresponding control cells were cultured in selection media (standard media according to above supplemented with 0.5–2 µg/mL puromycin).

RITA and Nutlin-3 were purchased from Cayman Chemical Company and Sigma-Aldrich, respectively, and SL-176 and SPI-001 were synthesized as described previously [49,51]. RITA and Nutlin-3 were dissolved in DMSO (Sigma-Aldrich), while SL-176 was dissolved in a mix of DMSO (33%) and ethanol (67%). Further dilutions were made in Opti-MEM or PBS. The DMSO concentration did not exceed 1% v/v in any experiment. For the in vivo studies, SL-176 was dissolved in a mixture of DMSO (33%) and ethanol (67%) and further diluted in sodium chloride 0.9%.

### 4.2. Short Hairpin RNA (shRNA)

For the transfections, cells were seeded in 6-well plates, left to attach and transfected using Lipofectamine 2000 (Thermo Fisher Scientific) with 4 µg of four pre-designed shRNAs (GIPZ Lentiviral) targeting human *PPM1D* (172_0502-F-1 (Clone ID: V2LHS_262759), 172_0556-D-7 (Clone ID: V2LHS_27794), 172-0447-C-2 (Clone ID: V2LHS_27798) and 172_0496-G-2 (Clone ID: V2LHS_262763), Dharmacon) and non-silencing pGIPZ Lenti Control shRNA (#RHS4346, Dharmacon). Cells were incubated for 6 h in the transfection medium, which was then replaced with corresponding culture medium. After 24–48 h, cells were subjected to further analyses. For generating stable transfections, cells were grown in selective medium (0.5–2 µg/mL puromycin selection).

### 4.3. Viability Assays

For evaluation of the cytotoxic effect on cell viability, we used the colorimetric formazan-based assay WST-1 (Roche), according to the manufacturer’s description. Briefly, cells were seeded into 96-well plates (5000–10,000 cells/well), incubated overnight and treated with drugs the following day. After 72 h, WST-1 reagent was added and absorbance was measured at 450 nm. All concentrations were tested in triplicate. The mean out of usually three or more independent experiments is reported.

To determine colony formation, 100 cells/well in the non-exposure experiments and 300 cells/well in the irradiation experiments were seeded in 60 mm cell+ culture plates (Sarstedt, Sweden) and allowed to attach for 24 h before exposure to ionizing radiation (Cobolt^60^ source) at 2 or 4 Gy, when applicable. After 10–14 days of incubation in medium, cells were washed, fixed in formaldehyde (4%), stained with Giemsa (Gibco, BRL Solna, Sweden) and colonies (1 clone > 50 cells) with 50% plate efficiency were manually counted. The mean out of at least three experiments is reported.

Cell viability after *PPM1D* silencing was assessed by the trypan blue exclusion assay. In brief, cells (4.4 × 10^4^ MEB-MED8A cells/well and 2.5 × 10^4^ SK-N-BE(2) cells/well, respectively) were seeded and transfected in six-well plates, three wells for each time point per cell line and transfection group, and cultured for six days. Cells were then stained with 0.4% trypan blue (GIBCO, BRL) and viable (unstained) cells were counted daily to determine the total number of living cells. The mean out of three experiments is reported.

### 4.4. Irradiation of Human Cancer Cell Lines

SK-N-BE(2), SH-SY5Y, DAOY, Med8a and MCF-7 cells were seeded into six-well cell culture plates (300,000–500,000 cells/well) in standard medium with 10% FBS and allowed to attach overnight, with exception for Med8a cells growing in suspension. Prior to treatment, 60–80% confluency was observed. Medium was removed and replaced with OptiMEM containing a SL-176 concentration equivalent to the corresponding IC_50_ value for each cell line (0.5–1.3 µM). Med8a cells were directly seeded in OptiMEM containing SL-176 at IC_50_. After 1h incubation, cells were irradiated with 4 Gy (Cobolt^60^ source) while kept on ice, after which incubation at 37 °C continued for the time indicated (0, 4, 8, or 24 h). Cells were harvested using Cell Dissociation Solution Non-enzymatic (Sigma-Aldrich). For the irradiation of the stably transfected SK-N-BE(2) neuroblastoma cell lines (*PPM1D* shRNA and control shRNA), 500,000 cells/well were seeded into six-well cell culture plates as described above, allowed to attach overnight and irradiated with 2 Gy and 4 Gy, respectively, and harvested after 48 and 72 h. SK-N-BE(2) non-transfected cells were treated with 40 mM cisplatin and used as a positive control for double-strand DNA breaks.

### 4.5. Western Blot

Protein extraction from cell lysate was performed in RIPA buffer (25 mM Tris (pH 7.8), 2 mM EDTA, 20% glycerol, 0.1% Nonidet P-40 (NP-40), 1 mM dithiothreitol), supplemented with protease inhibitor cocktail (Roche Diagnostic, Basel, Switzerland) and phosphatase inhibitor cocktail 1 (Sigma-Aldrich Solna, Sweden). The protein concentration was measured using Bradford protein assay (Bio-Rad Hercules, CA, USA). A total of 50 µg of protein was separated by SDS-PAGE, transferred to nylon membranes (Millipore Inc., Sundbyberg, Sweden) and incubated with antibodies against phosphorylated and total ATM (350 kDa); Wip1 (67 kDa); phosphorylated and total Chk1 (56 kDa) and Chk2 (62 kDa); phosphorylated and total p53 (53 kDa); phosphorylated and total p38 (43 kDa); total p21 (21 kDa); and ɣ-H2AX and H2AX (17 kDa). GAPDH (36 kDa) or β-tubulin (51 kDa) were used as internal controls. Anti-rabbit IgG conjugated with horseradish peroxidase (Cell Signaling) was used for secondary detection and Pierce Super Signal (Pierce) for chemiluminescent visualization. For a full list of antibodies, see Supplementary Table S4.

### 4.6. Quantitative Real-Time RT-PCR Analyses

The mRNA expression levels were quantified using TaqMan^®^ technology on an ABI PRISM 7500 sequence detection system (PE Applied Biosystems). Primers were selected from the Assay-on-Demand products (Applied Biosystems), including human PPM1D (Hs00186230_m1), and 18S ribosomal RNA (Hs99999901_s1). All gene expression assays were designed with an FAM reporter dye at the 5′ end of the TaqMan MGB probe, and a non-fluorescent quencher at the 3′ end of the probe. High-capacity RNA-to-cDNA kit (Applied Biosystems) was used to synthesize cDNA from 100 ng of RNA per sample. The PCR reaction was performed in a total reaction volume of 25 µL containing 1 × TaqMan^®^ Universal PCR Master Mix, 1 × TaqMan^®^ Gene Expression Assays (Applied Biosystems) and 10 µL of cDNA from each sample as a template, in MicroAmp optical 96-well plates covered with MicroAmp optical caps (Applied Biosystems). Firstly, samples were heated for 2 min at 50 °C and then amplified for 40 cycles of 15 s at 95 °C and 1 min at 60 °C. A standard curve was generated for relative quantification with cDNA synthesized from 1 μg RNA of the cell lines combined. For every sample, the amount of target mRNA was normalized to the standard curve and normalized to 18S ribosomal RNA expression. All experiments included a no template control and were performed in triplicate.

### 4.7. CRISPR-Cas9 Loss of Function Screening

The DepMap Public CRISPR (Avana) 18Q3 gene dependency dataset including 485 cancer cell lines (whereof 15 neuroblastoma and 7 medulloblastoma cell lines) as well as mutation call dataset was downloaded from the Broad Institute Cancer Dependency Map (https://depmap.org/portal/, 18Q3, accessed on 5 September 2018) and used for analysis of *PPM1D* and *TP53* genetic vulnerabilities [83]. Visualization and analysis of enriched functional processes associated with *TP53* dependency was performed in the Search Tool for the Retrieval of Interacting Genes/Protein (STRING) database [84].

### 4.8. Flow Cytometry

Phosphorylation of H2AX was assayed with Alexa Fluor 647-conjugated anti-phospho-H2AX (2F3, Biolegend, San Diego, CA, USA) at 24 and 72 h on cells that were transfected with *PPM1D*-shRNA 172_0502-F-1 and control shRNA, respectively. A minimum of 10,000 events were recorded on Becton–Dickinson FACSCalibur or LSR II flow cytometers (BD Biosciences, San Jose, CA, USA). Data analysis was performed using the Cell Quest software.

### 4.9. Ethical Permits

The animal experiments were approved by the regional ethics committee for animal research in Northern Stockholm, appointed by and under the control of the Swedish Board of Agriculture and the Swedish Court (ethical approvals N304/08 and N391/11). All animal experiments were in accordance with national regulations (SFS 1988:534, SFS 1988:539, and SFS 1988:541).

### 4.10. Xenograft Studies

Immunodeficient nude mice (female 4–6 weeks old, NMRI-nu/nu, Scanbur, Stockholm, Sweden) were used for xenograft studies. The mice were kept under specific pathogen-free conditions at a maximum of six individuals per cage and given sterile water and food ad libitum. All mice were treatment-naïve at the start of the experiment.

Under general anesthesia, each mouse was injected subcutaneously on the rear flank with 10 × 10^6^ SK-N-BE(2) neuroblastoma cells or 17 × 10^6^ DAOY medulloblastoma cells. In the knockdown experiment, mice were inoculated bilaterally with 5 × 10^6^ SK-N-BE(2) cells (clone d) that were knocked down for *PPM1D* (*n* = 8) and SK-N-BE(2) control cells (clone C) that were transfected with non-silencing shRNA (*n* = 15), respectively, and followed until the tumor reached 0.1 mL.

In the drug treatment experiments, mice with SK-N-BE(2) xenografts were randomly assigned to three different treatment groups when the tumor reached ≥0.1 mL. For twelve days, mice received either daily intraperitoneal injections (i.p.) of SL-176 at 3 mg/kg (*n* = 8) or 0.5 mg/kg (*n* = 6), or no treatment (*n* = 6). The mean tumor volume at the start of treatment was 0.115 mL.

Mice bearing DAOY xenografts were randomly divided into two different groups, and treatment commenced at tumor volume ≥ 0.12 mL. Mice received either 3 mg/kg SL-176 as daily i.p. injection for 21 days (*n* = 8), or no treatment (*n* = 7). The mean tumor volume at the start of treatment was 0.124 mL.

In all xenograft-bearing mice, tumors were measured by caliper every day and the animals were monitored for signs of toxicity including weight loss. The tumor volume was estimated as (width)^2^ × length × 0.44. At sacrifice, tumors were dissected and either frozen or fixed in formaldehyde, for subsequent analyses.

### 4.11. Immunohistochemistry of Xenograft Tumors

Formalin-fixed and paraffin-embedded tissue sections were deparaffinized in xylene and graded alcohols, hydrated and washed in phosphate-buffered saline (PBS). After antigen retrieval in sodium citrate buffer (pH 6) in a microwave oven, endogenous peroxidase was blocked by 0.3% H_2_O_2_ for 15 min. Sections were incubatedovernight at 4 °C with primary antibody phosphor-histone H2A.X (Ser139) (Cell Sinalling), cleaved caspase-3 (Asp175) (Cell signaling) or Ki67 (SP6, Neomarkers, CA, USA). Anti-rabbit-horseradish peroxidase (HRP) was used as secondary antibody. SignalStain Boost kit (Cell Signalling) was used for detection. Matched isotype controls were used as a control for non-specific staining.

### 4.12. Statistical Analysis

Statistical analyses were performed with GraphPad Prism software (GraphPad Software, San Diego, CA, USA). The IC_50_ values (inhibitory concentration 50%) were determined from log concentrations–effect curves using non-linear regression analysis. T test was used to compare means between two groups, and for comparison of three or more groups, one-way ANOVA followed by Bonferroni multiple-comparisons post hoc test were used. Paired analysis was performed with repeated measures ANOVA or, when values were missing, on mixed-effect analysis with Bonferroni multiple-comparisons post hoc test. Survival analysis was examined with log-rank test, and Fisher’s test was used to test significance of association between the two categories. Correlations were assessed with Pearson test/Spearman non-parametric test. *p* < 0.05 was considered significant and all tests were two-sided. Survival curves were calculated using the Kaplan–Meier method.

## 5. Conclusions

Overexpression of *PPM1D* is an important tumorigenic factor in medulloblastoma and neuroblastoma, as these tumor cells are highly dependent on high levels of *PPM1D* for their survival. Compounds that inhibit the activity of WIP1 suppress both neuroblastoma and medulloblastoma growth in mouse xenograft models. Unfortunately, all the WIP1 inhibitors described to date remain non-viable clinically, either due to poor solubility or because of poor pharmacokinetic properties of the compounds in vivo. Since the structure of WIP1 is still unknown, inhibitors of WIP1 have to a large extent been identified through high-throughput screening of chemical libraries [19]. Hence, more specific inhibitors of WIP1 with better bio-availability need to be developed in order to accurately demonstrate the potential of inhibiting WIP1 in patients.

## Data Availability

The data presented in this study are available on request from the corresponding author. The data are not publicly available due to privacy.

## Supplementary Materials

The following are available online at https://www.mdpi.com/article/10.3390/cancers13236042/s1, Figure S1: Chromosome 17q copy number gains in medulloblastoma cell lines. Comparative genomic hybridization (CGH) array of five medulloblastomas and one supratentorial primitive neuroectodermal tumor cell line PFSK-1, illustrated as a blue line in single chromosome view. The red and green lines show the strongest and weakest allele intensity, respectively for each cell line. Arrows indicate gain of chromosome 17q. For allele-specific intensity calculations using AsCNAR algorithm, see [42], Figure S2: *PPM1D* knockdown increases H2AX phosphorylation. γH2AX was measured with flow cytometry 24 and 72 h after transfection with shRNA against *PPM1D* or control shRNA, Figure S3: The WIP1 phosphatase inhibitor SL-176 suppresses neuroblastoma and medulloblastoma viability. A. Dose–response curves for cell viability after 72 h of treatment with three WIP1 inhibitors; SL-176, SPI-001 or CCT007093, in neuroblastoma cell lines and breast cancer cell line MCF-7. Cell viability was determined using WST-1 assays. Data represent the mean ± S.E.M. of at least three experiments. B. Dose–response curves for cell viability after 72 h of treatment with SL-176, RITA or Nutlin-3 in neuroblastoma cell lines, medulloblastoma cell lines, the sPNET cell line PFSK-1 and the breast cancer cell line MCF-7. The fibroblast cell line MRC-5 and the murine neural progenitor cell line C17.2 were used as non-tumorigenic controls for drug toxicity. Cell viability was determined with WST-1. Data represent the mean ±S.E.M. of at least three experiments, Figure S4: Original western blots (Figure 3). Table S1: *TP53* wild-type neuroblastoma cell lines displayed a higher genetic dependency of *PPM1D* as compared to *TP53* mutated cell lines, Table S2: IC50 values for WIP1 inhibitors, RITA and Nutlin-3, Table S3: *TP53* mutational status in the cell lines used in the study, Table S4: List of antibodies used for Western blotting.
