# Supplementary material for: PPM1D Is a Therapeutic Target in Childhood Neural Tumors
Source: Cancers (Basel). 2021 Nov 30;13(23):6042. doi: 10.3390/cancers13236042 (PMC8657050; doi:10.3390/cancers13236042)
Supplement: Supplementary file 1 [file cancers-13-06042-s001.zip › Supplementary files/Supplementary Figure S4.pptx]

## Slide 1
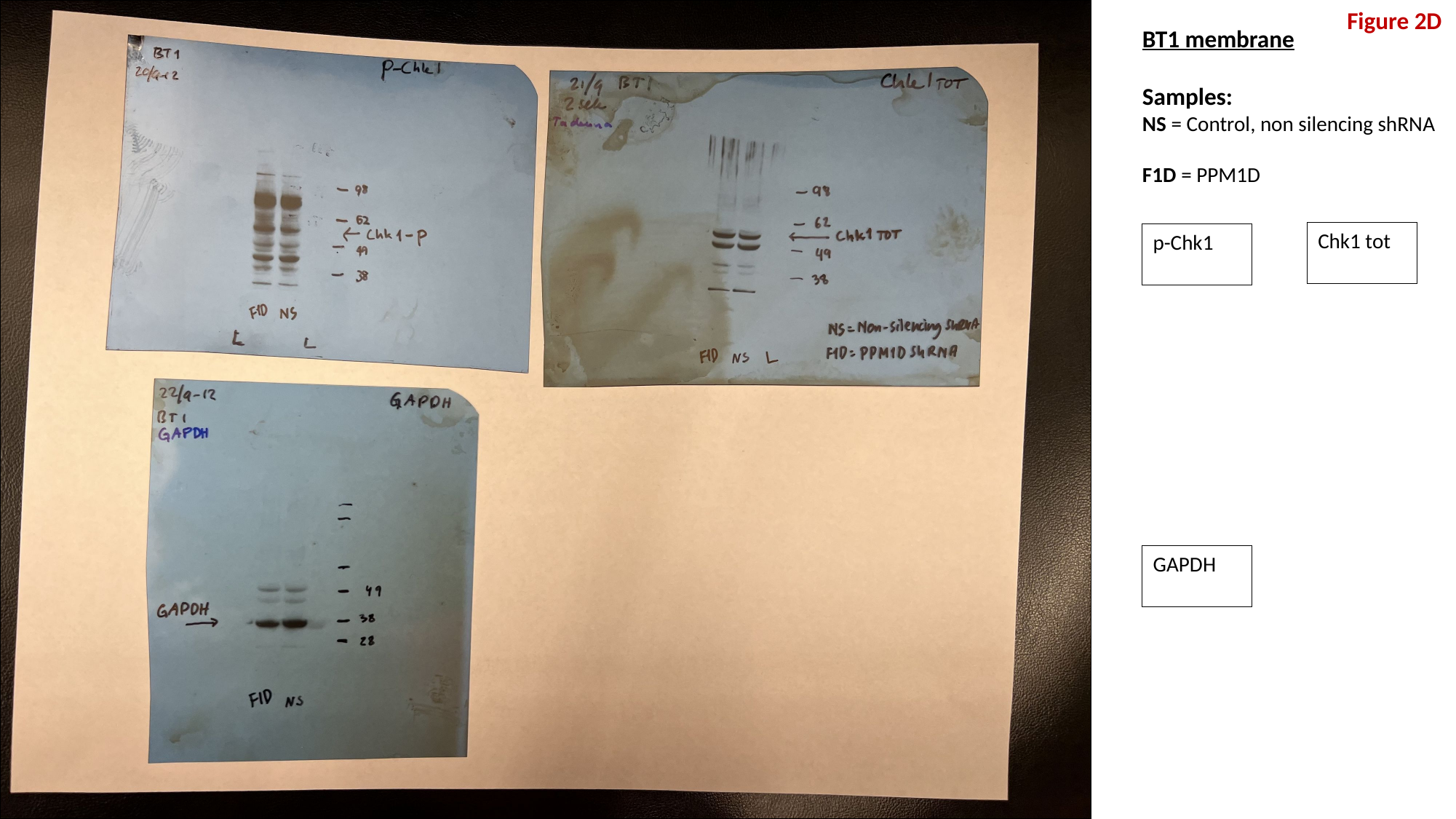

Figure 2D
BT1 membrane
Samples:
NS = Control, non silencing shRNA
F1D = PPM1D
Chk1 tot
p-Chk1
GAPDH

## Slide 2
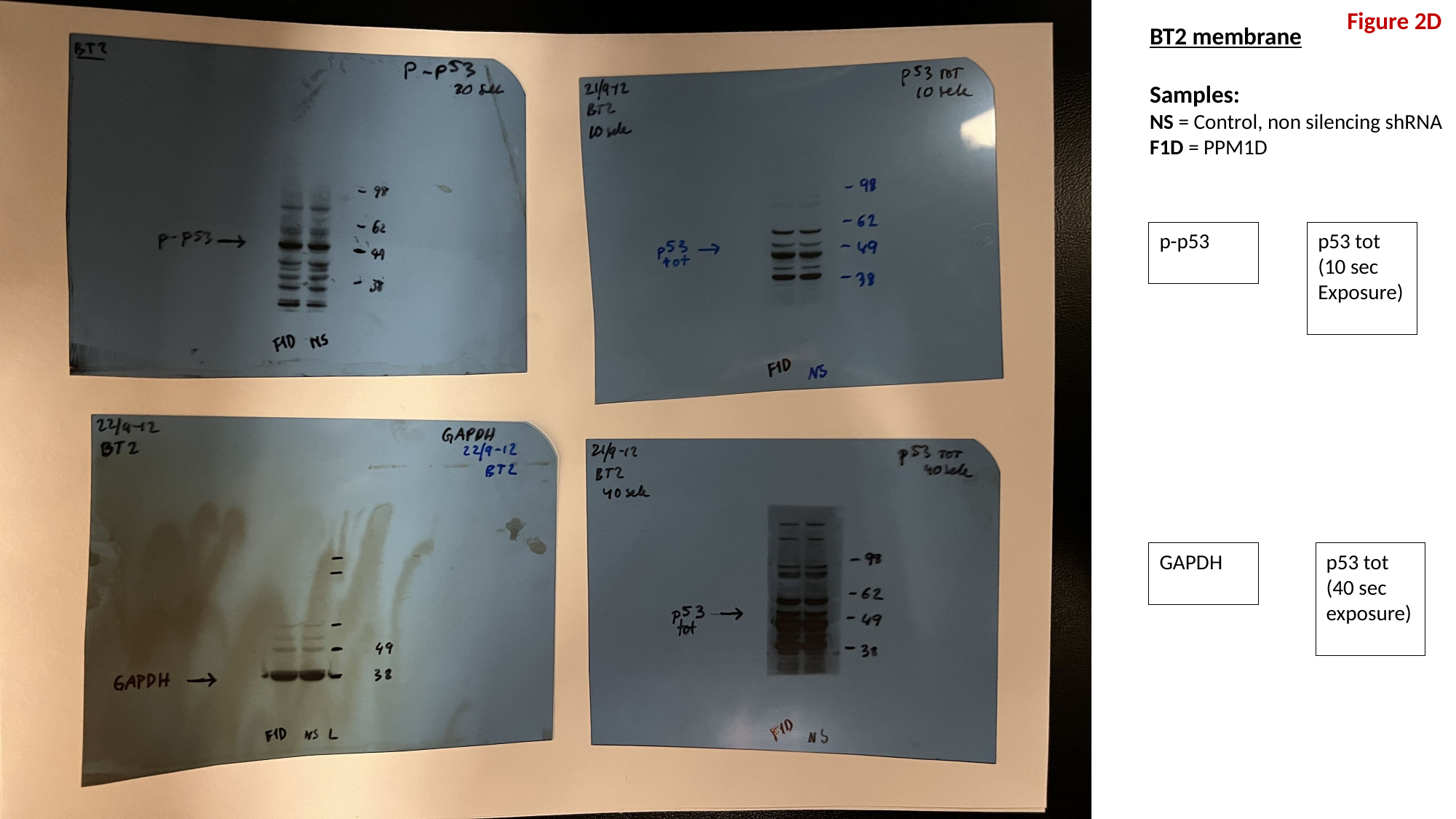

Figure 2D
BT2 membrane
Samples:
NS = Control, non silencing shRNA
F1D = PPM1D
p-p53
p53 tot
(10 sec
Exposure)
GAPDH
p53 tot
(40 sec exposure)

## Slide 3
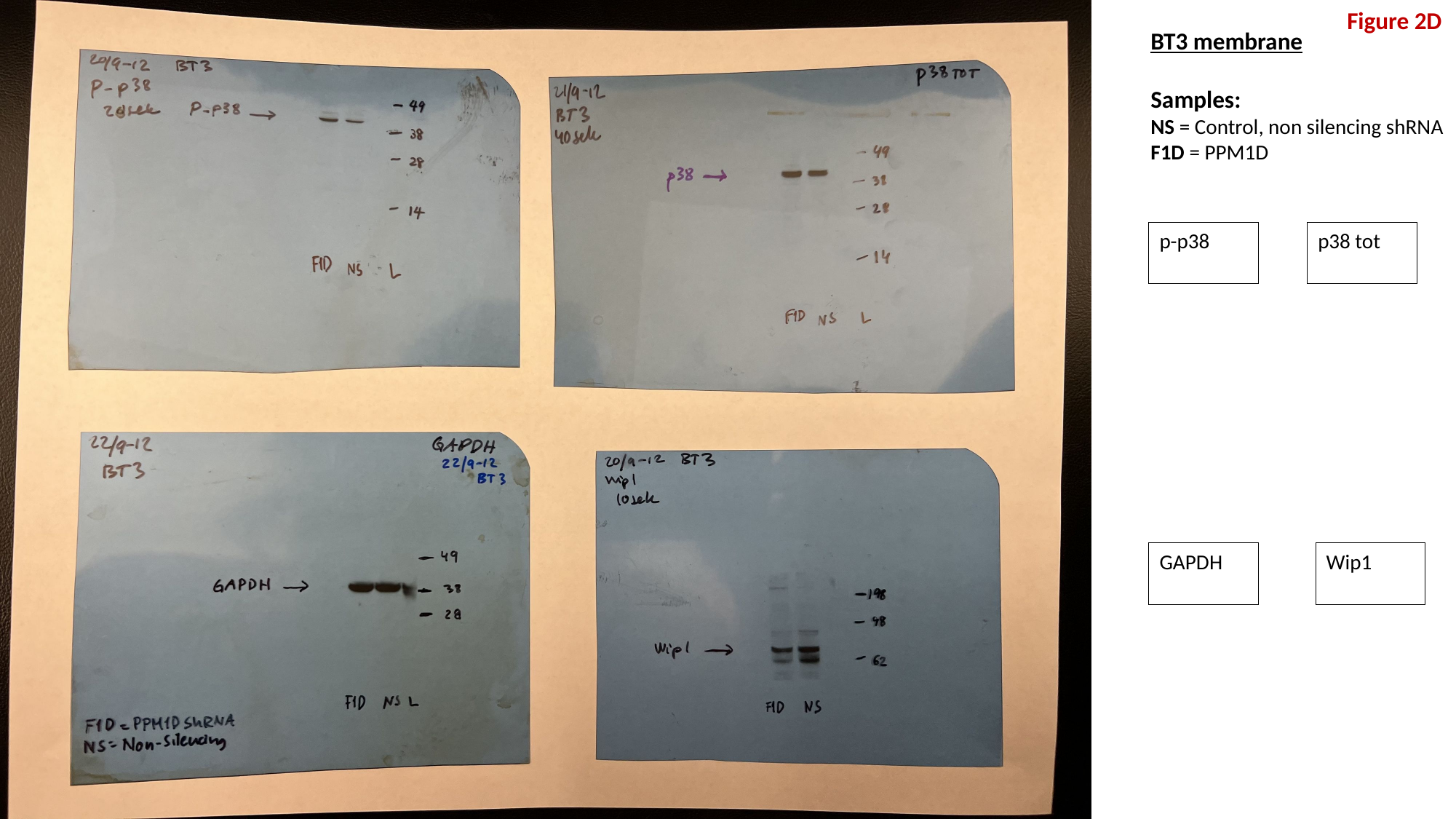

Figure 2D
BT3 membrane
Samples:
NS = Control, non silencing shRNA
F1D = PPM1D
p-p38
p38 tot
GAPDH
Wip1

## Slide 4
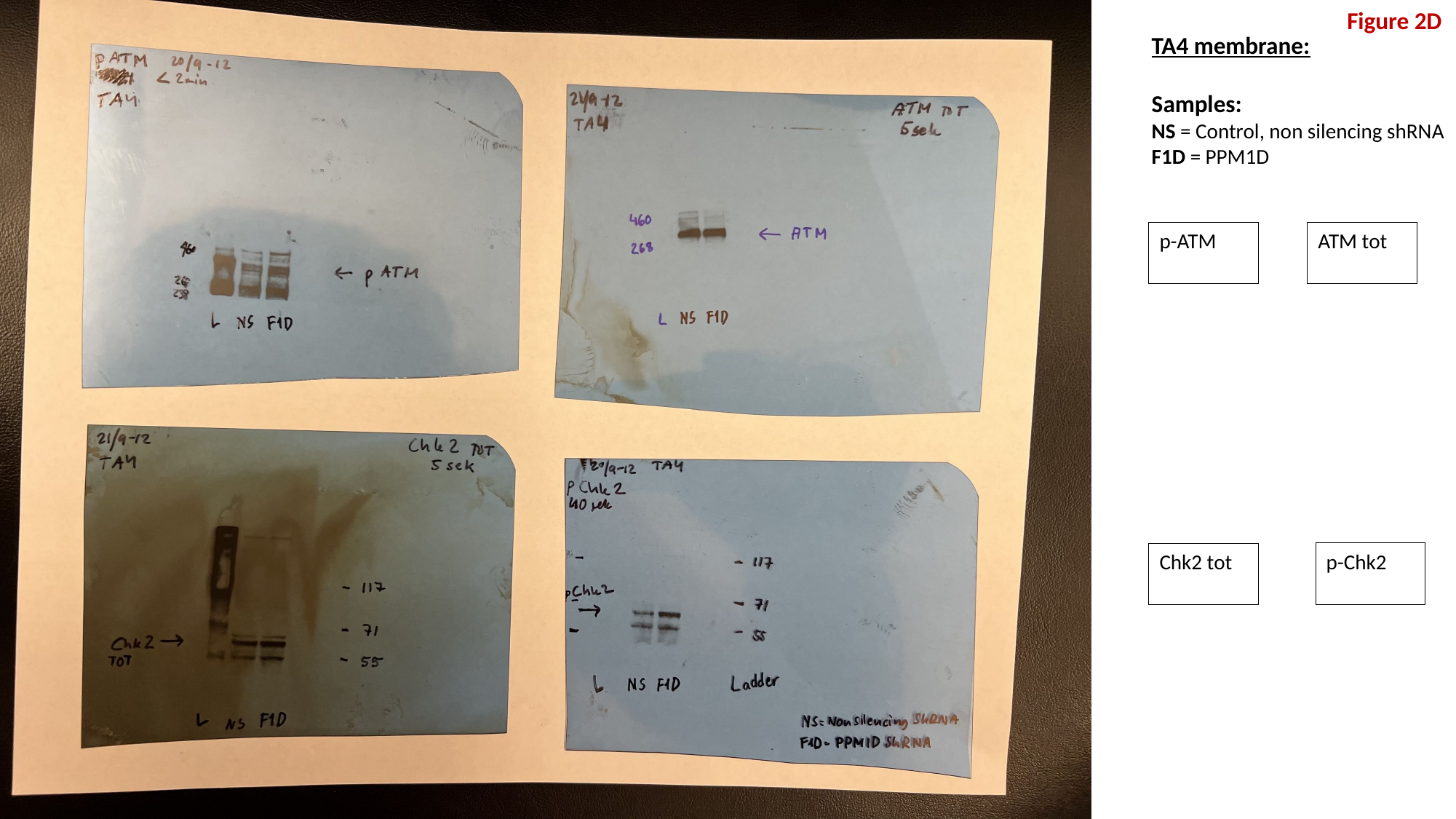

Figure 2D
TA4 membrane:
Samples:
NS = Control, non silencing shRNA
F1D = PPM1D
ATM tot
p-ATM
p-Chk2
Chk2 tot

## Slide 5
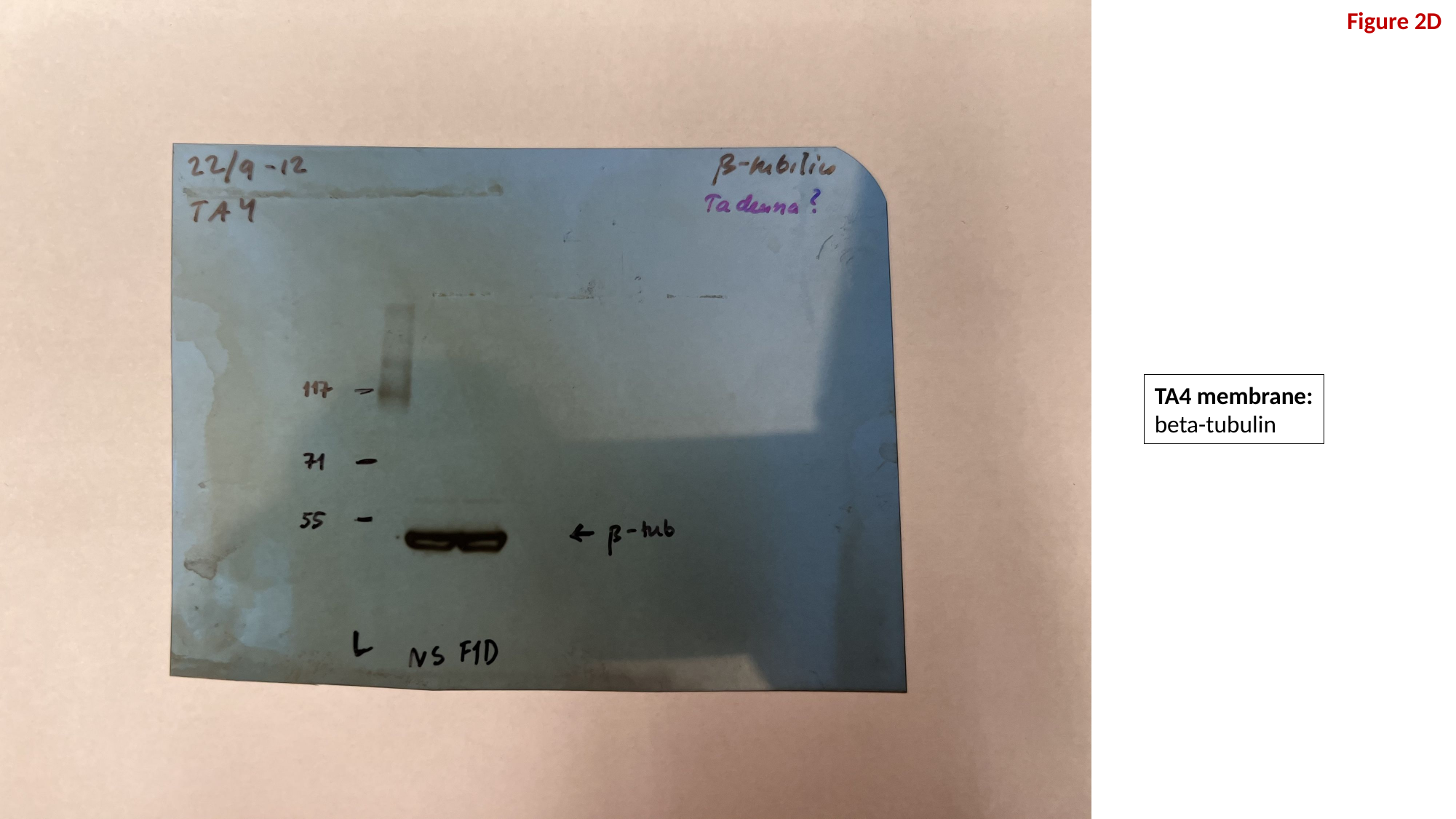

Figure 2D
TA4 membrane:
beta-tubulin

## Slide 6
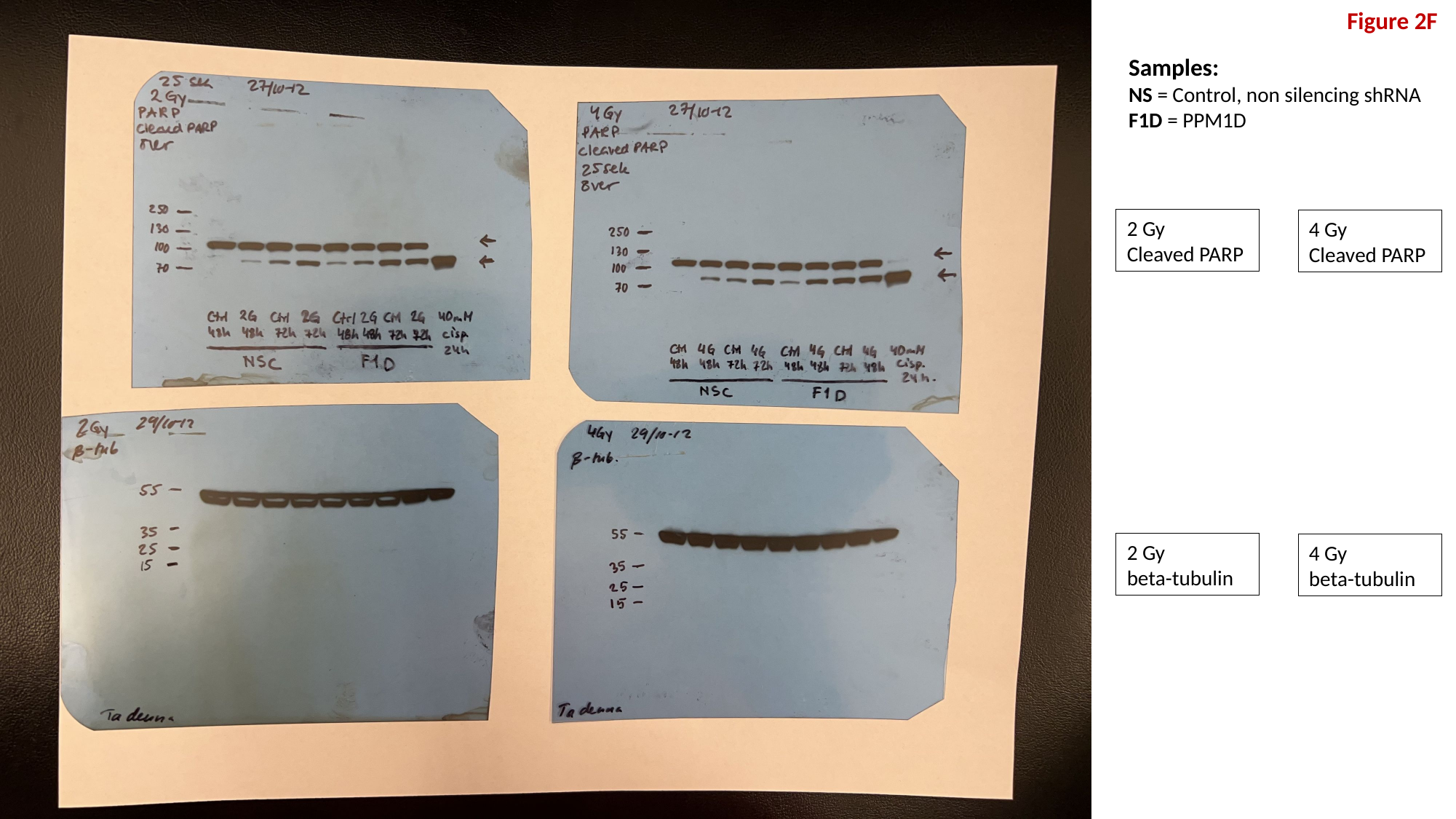

Figure 2F
Samples:
NS = Control, non silencing shRNA
F1D = PPM1D
2 Gy
Cleaved PARP
4 Gy
Cleaved PARP
2 Gy
beta-tubulin
4 Gy
beta-tubulin
