# Supplementary material for: PPM1D Is a Therapeutic Target in Childhood Neural Tumors
Source: Cancers (Basel). 2021 Nov 30;13(23):6042. doi: 10.3390/cancers13236042 (PMC8657050; doi:10.3390/cancers13236042)
Supplement: Supplementary file 1 [file cancers-13-06042-s001.zip › Supplementary files/Supplementary Figures-Milosevic Revision.pptx]

## Slide 1
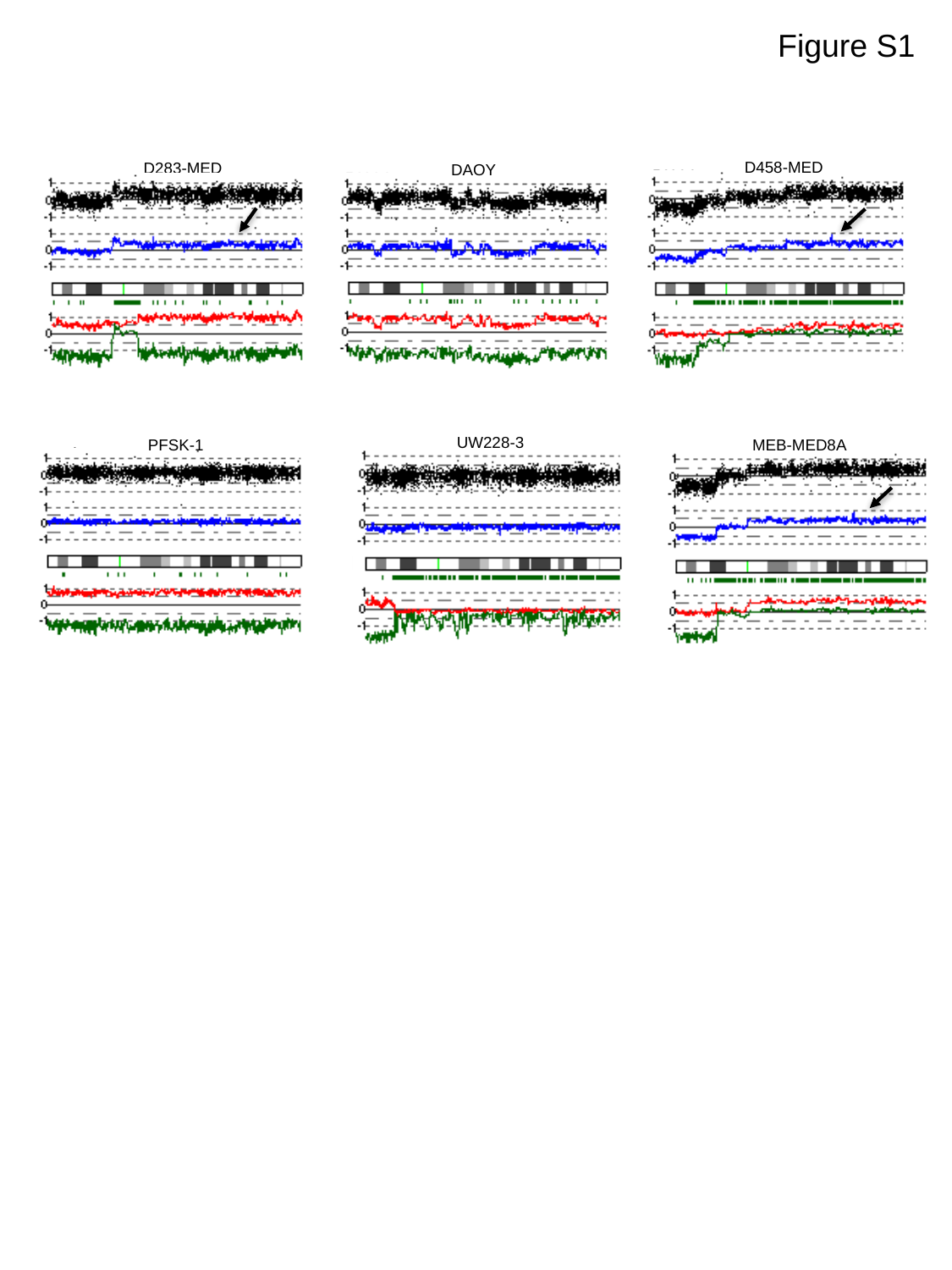

Figure S1
D458-MED
D283-MED
DAOY
UW228-3
MEB-MED8A
PFSK-1

## Slide 2
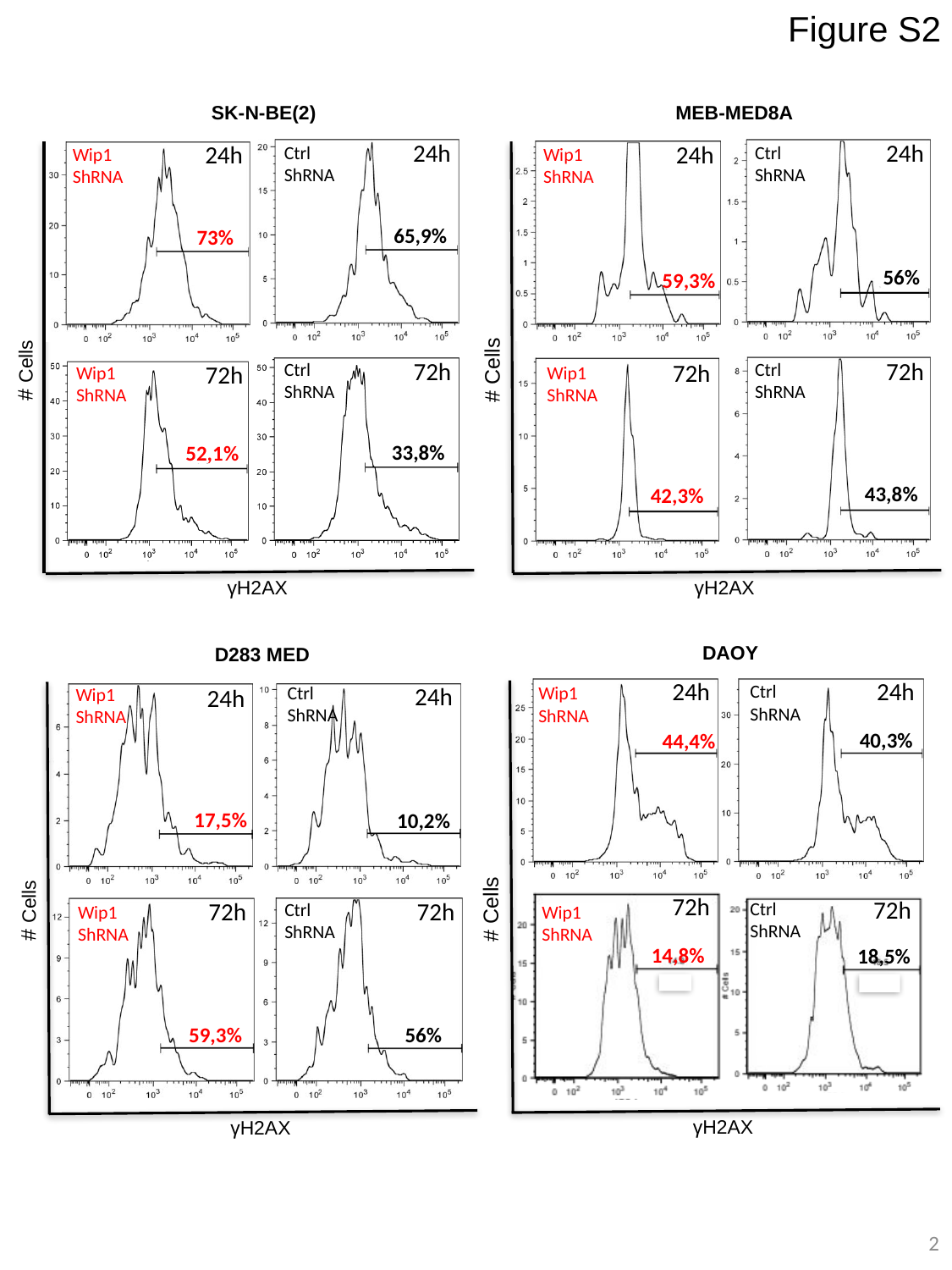

Figure S2
SK-N-BE(2)
65,9%
73%
72h
33,8%
52,1%
# Cells
γH2AX
Ctrl
ShRNA
Wip1
ShRNA
Ctrl
ShRNA
Wip1
ShRNA
MEB-MED8A
# Cells
γH2AX
24h
56%
59,3%
Ctrl
ShRNA
Wip1
ShRNA
Ctrl
ShRNA
Wip1
ShRNA
43,8%
42,3%
24h
72h
72h
72h
24h
24h
DAOY
Ctrl
ShRNA
Wip1
ShRNA
# Cells
γH2AX
40,3%
44,4%
Ctrl
ShRNA
Wip1
ShRNA
14,8%
18,5%
D283 MED
Ctrl
ShRNA
24h
17,5%
10,2%
Wip1
ShRNA
# Cells
γH2AX
72h
59,3%
56%
Ctrl
ShRNA
Wip1
ShRNA
24h
24h
24h
72h
72h
72h
72h
2

## Slide 3
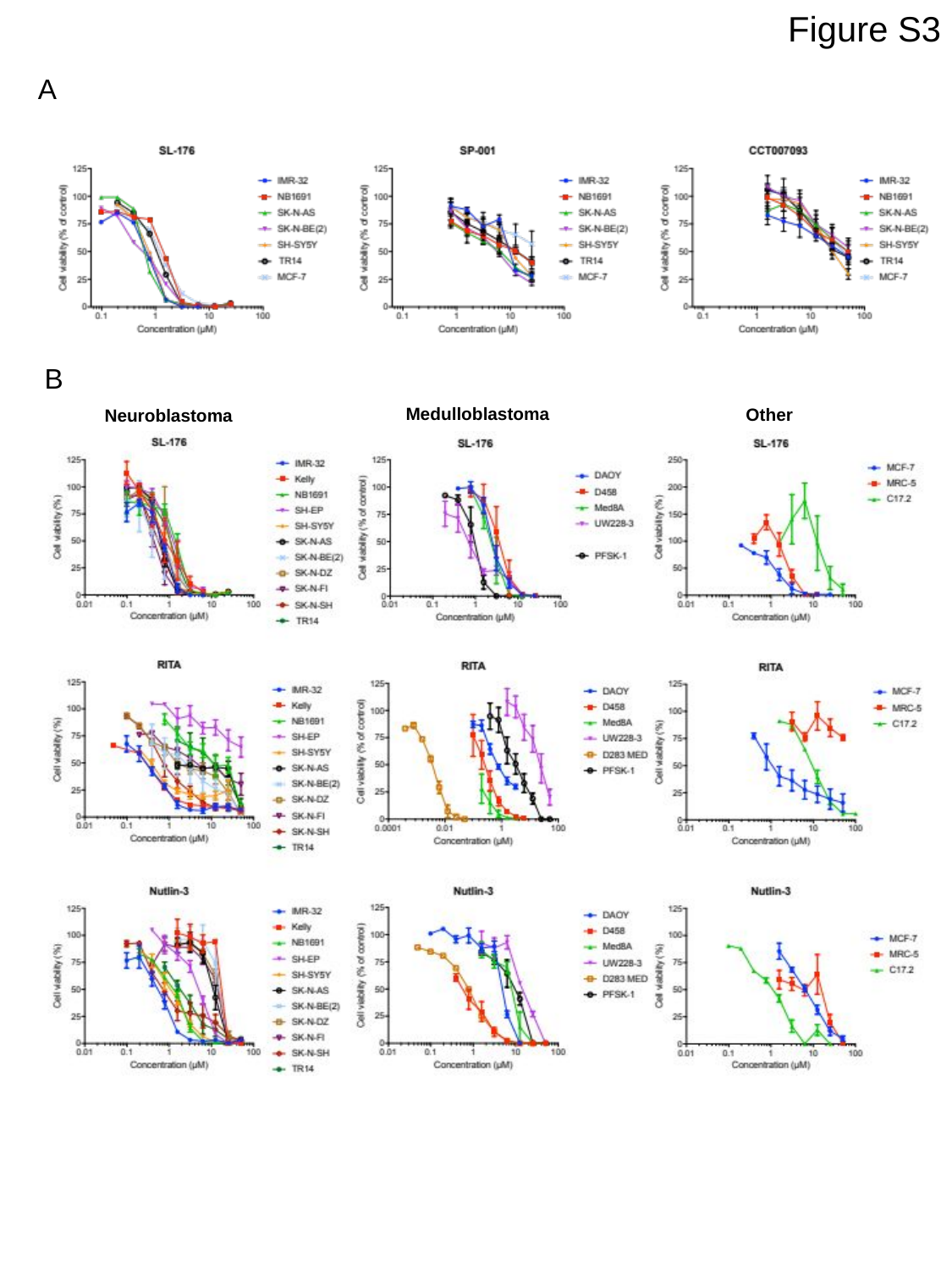

Figure S3
A
B
Medulloblastoma
Other
Neuroblastoma
